# Bubble Trouble: Quantifying the Effects of Bubbles on the Electrochemical Interface

**DOI:** 10.1021/acscatal.5c00144

**Published:** 2025-04-04

**Authors:** Anja Logar, Dževad K. Kozlica, Ožbej Vodeb, Miran Gaberšček, Nejc Hodnik, Dušan Strmčnik

**Affiliations:** † National Institute of Chemistry, Department of Materials Chemistry, Hajdrihova 19, 1000 Ljubljana, Slovenia; ‡ University of Nova Gorica, Graduate School, Vipavska 13, 5000 Nova Gorica, Slovenia; § University of Maribor, Faculty of Chemistry and Chemical Engineering, Smetanova ulica 17, 2000 Maribor, Slovenia; ∥ Jozef Stefan International Postgraduate School, Jamova 39, 1000 Ljubljana, Slovenia; ⊥ University of Ljubljana, Faculty of Chemistry and Chemical Technology, Večna pot 113, 1000 Ljubljana, Slovenia; # Institute of Metals and Technology, Department of Physics and Chemistry of Materials, Lepi Pot 11, 1000 Ljubljana, Slovenia

**Keywords:** oxygen evolution reaction, hydrogen evolution reaction, bubbles, rotating disk electrode, uncompensated resistance

## Abstract

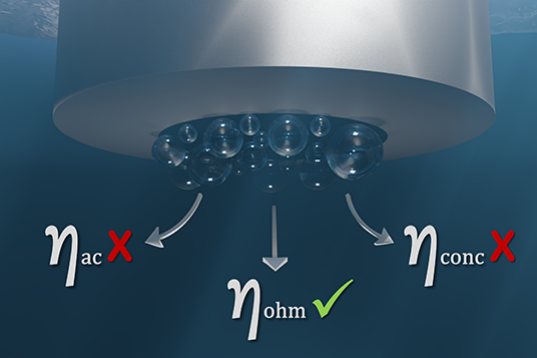

The accumulation of electrochemically produced bubbles is inevitable in gas-evolving reactions and can induce potential losses by theoretically increasing activation, concentration, and ohmic overpotentials. These effects are often either overstated or completely neglected in the literature, which complicates the accurate analysis of experimental results for gas evolution reactions. This study systematically identifies and quantifies the overpotential losses induced by bubbles by combining experimental results for hydrogen (HER) and oxygen evolution reactions (OER), obtained using the rotating disk electrode (RDE) technique, with simulations based on a two-dimensional transmission line model. Our results show that ohmic overpotential is the primary cause of apparent activity loss due to bubbles in RDE. This effect leads to catalyst activity misestimates exceeding 2 orders of magnitude, and Tafel slope errors of 100% at higher currents if left uncorrected. By identifying these effects, this work provides a robust framework for mitigating inaccuracies and improving the characterization of electrocatalysts for gas evolution reactions.

## Introduction

Rotating disk electrode (RDE) has been successfully used in fundamental studies of different catalytic platforms ranging from well-defined single-crystal surfaces to thin films of nanoparticulated materials, used in real systems, e.g. fuel cells and electrolyzers.^1^ Moreover, it has been used in the study of a plethora of electrocatalytic reactions, including gas-producing reactions such as hydrogen (HER) and oxygen evolution reactions (OER). The electrochemistry of gas evolution reactions is inevitably interlinked with the formation of bubbles at the electrochemical interface. The nucleation from the supersaturated electrolyte solution and the dynamics of their growth and detachment are widely investigated processes, which have been also gaining increasing interest in the electrocatalytic community.^2−7^ On one hand, this interest stems from the desire to mitigate their adverse effects on various electrochemical systems. On the other hand, however, it stems from the observed discrepancies between the catalytic behavior measured in RDE and real systems which were also attributed to a different accumulation dynamics of the bubbles produced within the catalyst layer.^8^ The latter has led even to several publications questioning the general validity of RDE electrocatalytic data and its transferability to real systems such as MEA.^9,10^

On the other side of the spectra, however, there is still a limited attention given to the challenges that bubbles create for the accurate measurement of the electrocatalytic activity of materials. This can lead to inaccurate conclusions when comparing the intrinsic catalytic properties of the materials,^11^ conclusions regarding reaction mechanisms^4^ as well as potential misinterpretations of phenomena such as the effect of an external field on the catalysis of gas-evolving reactions.^12^ All of these issues fall well within the effects bubbles can exert on the electrochemical interface.

Based on the extensive research in this area, three components of the overpotential losses influenced by the existence of bubbles have been recognized in the literature and summarized in eq 1.^13,14^
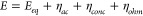
1where *E*_*eq*_ is the equilibrium potential for a given reaction. It has been reported that when bubbles start to grow, they accumulate on the surface of the catalyst and increase the activation overpotential η_*ac*_ of the reaction by blocking the active surface area. Their dispersion in the electrolyte obstructs the ion migration and contributes to the increase in its ohmic resistance η_*ohm*_. After nucleation, they start to grow and essentially keep the solution next to the electrode saturated and thus affect the concentration overpotential η_*conc*_. While the effects of bubbles seem to be well understood, at least in theory, the quantification of their effect is to a large extent still missing, and therefore this knowledge is inadequately applied in practical examinations of catalysts for gas evolution reactions.

Herein, we employ a systematic approach to quantify the effects of bubbles through a combination of an innovative two- dimensional transmission line model and experimental observations in an RDE setup for two gas evolution reactions on three different catalyst platforms, namely OER on hydrous oxide covered-polycrystalline Ir disk, OER on Ir/C thin film and HER on Ni polycrystalline disk. We experimentally observe and quantify exclusively the effect of constriction of the conductive pathways to the interface by bubbles, i.e. the increase of the effective electrolyte resistance during the electrochemical measurements. To make a distinction to their effect on the resistance, we furthermore show the simulated effect of bubbles on activation and concentration overpotentials and discuss the observations reported in the literature. Finally, we propose strategies to mitigate RDE measurements of gas evolution reactions and show that as long as we have a quantitative understanding of the effects, we should neither fear nor disregard the presence of bubbles at the electrochemical interface in the RDE setup.

## Results and Discussion

**Figure 1:**
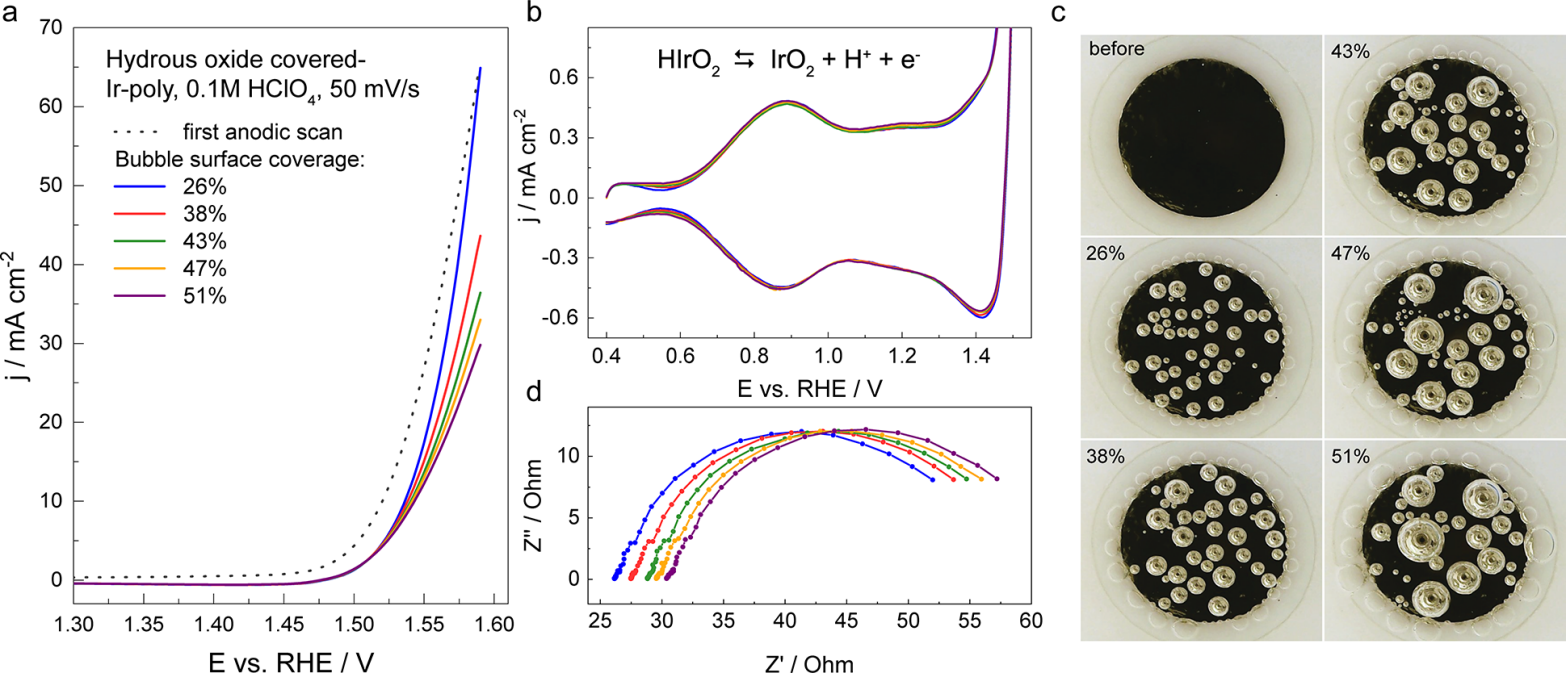
Electrochemical results obtained on electrochemically grown hydrous oxide covered-polycrystalline Ir RDE, measured in 0.1 M HClO_4_. a) Consecutive OER polarization curves with increasing bubble surface coverage. Cathodic scans (full lines) are shown as they more accurately correspond to the determined bubble coverage. First anodic scan is also shown (dashed line), that was measured on the initially bare surface which was progressively becoming more covered with oxygen bubbles. b) Cyclic voltammograms measured with a 50 mV/s scan rate, indicating no change in the available surface area of the disk with increasing bubble surface coverage, c) micrographs of the disk with different surface coverages taken after each CV and d) EIS recorded at 1.52 V after each cyclic voltammogram.

To distinguish and quantify the overpotential components arising from the accumulation of bubbles on the surface of RDE, as defined in eq 1, and thoroughly understand how their existence is manifested in the experimental results, we performed potentiodynamic measurements for OER on an electrochemically grown hydrous oxide covered- polycrystalline Ir RDE in acidic media. The electrochemical protocol consisted of five consecutive potential scans from the lower potential limit of 0.4 V to monitor the changes in the availability of the surface area of Ir to the upper potential limit 1.59 V to measure the OER polarization curve (Figure 1a, b). Each consecutive cycle resulted in an increasingly higher coverage of the disk with oxygen bubbles. To assess the percentage of the surface covered by bubbles after each excursion into an OER region, a digital microscope was placed under the working electrode and the changes on the surface of the electrode were recorded simultaneously with the electrochemical experiment (link to the videos can be found in the Supporting Information). Images taken after each cycle are shown in Figure 1c. Voltammetry was measured with a 95% *iR*-drop compensation as is common practice in the literature.^15^ After each scan, electrochemical impedance spectra (EIS) were recorded at 1.52 V, just above the OER onset potential (Figure 1d). To preserve the generated bubbles on the surface of the disk, we intentionally performed the experiments without rotating the electrode. A detailed description of the electrode preparation and experimental protocol can be found in the Supporting Information. The universality of our approach and findings was tested also on a thin film of powdered Ir-based catalyst for OER and polycrystalline Ni disk, used as a model catalyst for HER in alkaline media (additional discussion included in Supplementary Note 1).

As shown in Figure 1a, the apparent activity for the OER, expressed as the current density at a given potential is significantly decreasing (by a factor of ∼2 at 1.55 V) with the increasing coverage of the disk with oxygen bubbles (Figure 1a). Nevertheless, the electrochemical surface area remains practically unaffected by the bubble coverage, as can be seen from the overlapping voltammograms (Figure 1b) in the potential range 0.4–1.4 V with the main redox peak at approximately 0.87 V being attributed to the Ir valence change due to incorporation/removal of protons, normally used to calculate the electrochemical surface area (ECSA) of Ir.^16,17^ This seemingly counterintuitive observation can be explained by looking at the microscopic images in Figure 1c, where it is evident that due to their spherical shape, the bubbles are only minimally touching the electrode surface. To obtain relevant EIS spectra, the measurements were performed at the potential, where a notable OER current was recorded on one hand but was nevertheless not contributing significantly to further growth of oxygen bubbles. To acquire such impedance spectra in a wide frequency range from 100 kHz to 1 Hz under steady-state conditions, it was necessary to use a potentiostat that allowed a fast EIS measurement (approximately 20 s). The resulting Nyquist plots (Figure 1d) show a positive shift of the semicircles along the real axis with increasing surface coverage and a negligible change in their shape.

**Figure 2:**
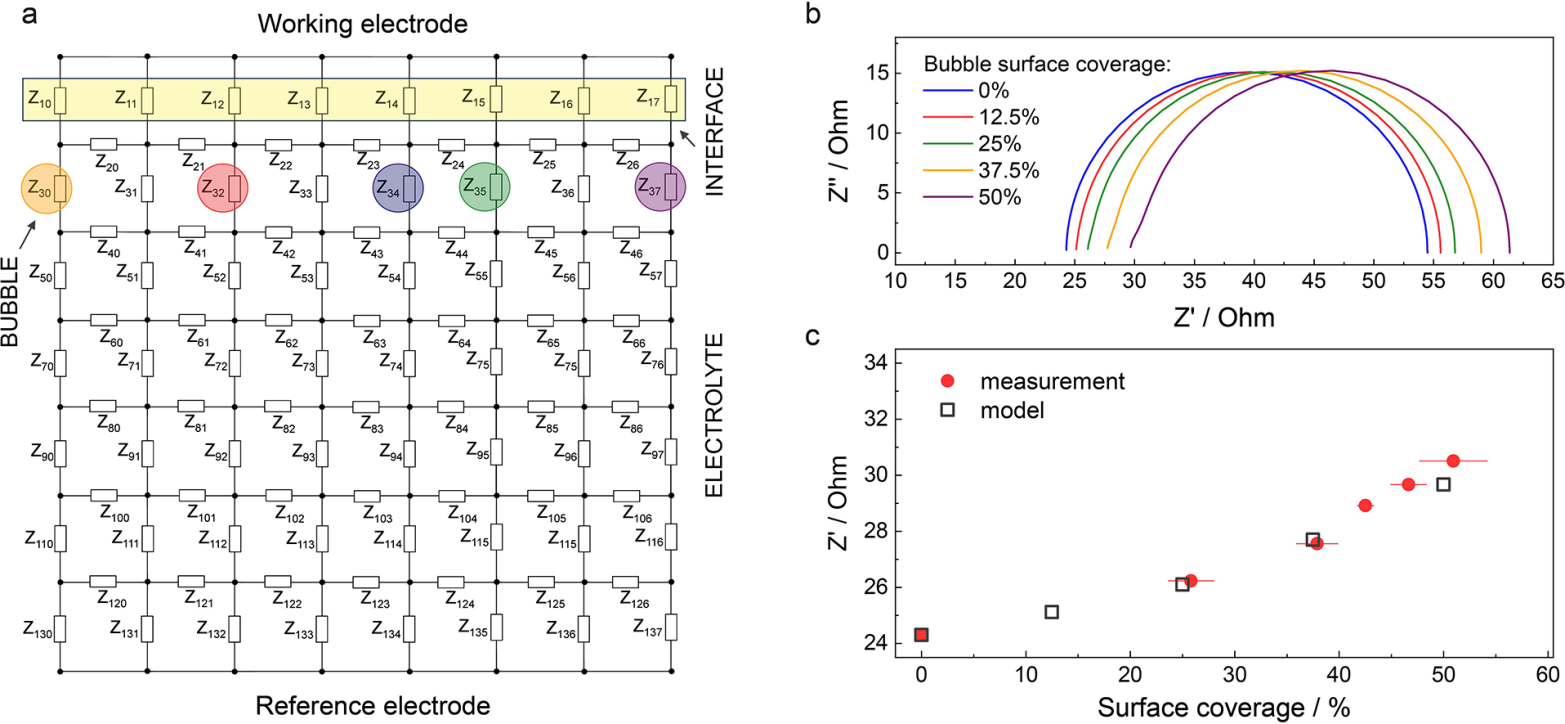
a) Transmission line model, used for the simulations of the EIS spectra, which includes both the electrolyte and reaction resistance with varying amounts of generated bubbles on the electrochemical interface, b) Nyquist plots, simulated based on the transmission line model for electrodes with varying coverages of the surface with bubbles, c) comparison of the model-predicted high frequency intercept along the *x*-axis with the experimentally obtained EIS results. The uncompensated resistance of electrolyte, i.e. at 0% coverage, used as an input parameter for the simulation of the EIS spectra was measured at a potential below the onset of OER, at 1.3 V.

To explain the measured trends, we used a two-dimensional transmission line model that takes into account the effects of bubbles on both ion conduction in the electrolyte and the reaction resistance at the catalyst surface (Figure 2a, detailed description in Supplementary Note 2). Based on the known bubble geometry and their spatial distribution obtained from the microscopic data (Figure 1c), we simulated the variation of the impedance spectra at different percentages of bubble coverage (Figure 2b). As mentioned above, the accumulation of bubbles notably shifts the high-frequency intercept of the *x*-axis of the Nyquist plot to higher values. This indicates that the bubbles mainly affect the uncompensated resistance, *R*_*u*_. Comparison of the measured *R*_*u*_ values with the values, extracted from the simulated spectra shows that the model is able to accurately predict the change in the spectra with the accumulation of bubbles on the surface of the catalyst (Figure 2c). As explained in the Supplementary Note 2, no curve fitting was used to create the model curve in Figure 2b.

With ohmic overpotential identified as an exclusive mechanism through which the bubbles cause the deactivation of the interface in all provided examples, we have to conclude that at least in the RDE setup the reported effect of the bubbles on activation and concentration overpotentials does not play any significant role.

**Figure 3:**
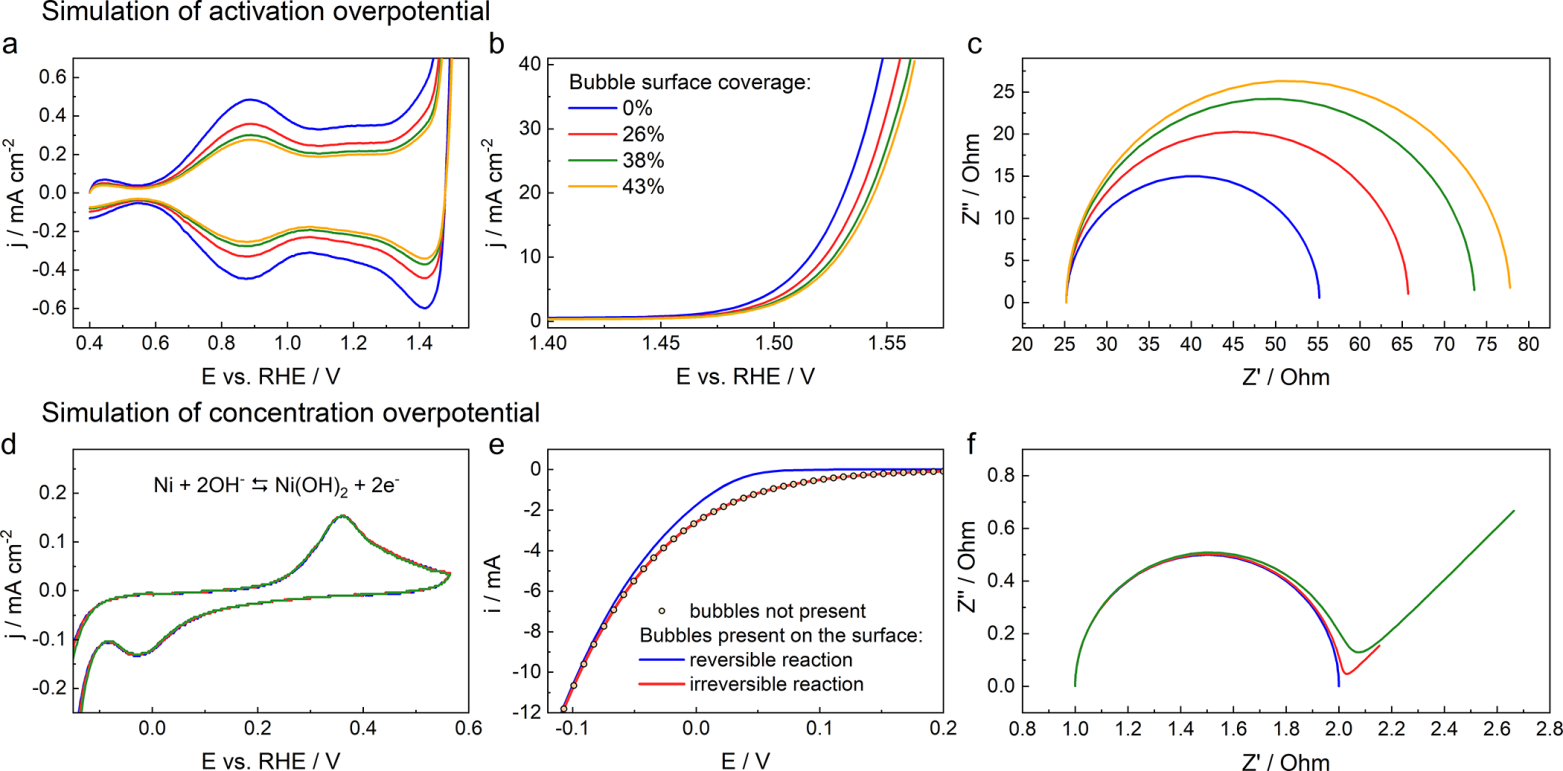
a-c: Simulation of the effect of the activation overpotential on the a) voltammetry, b) polarization curves and c) EIS spectra, for OER on polycrystalline Ir RDE in 0.1 M HClO_4_; d-f: Simulation of the effect of concentration overpotential on the d) voltammetry of polycrystalline Ni RDE in 0.1 M KOH, used as a model system for irreversible HER, e) polarization curves of reversible and irreversible HER and f) EIS spectra.

Although not possible to measure experimentally in the RDE setup, in Figure 3 we provide a simulation of the voltammetries, polarization curves and impedance spectra one would obtain, if the bubbles would affect the concentration or activation overpotential. This gives us a tool for identifying individual contributions to potential losses across the electrochemical interface due to bubbles.

First, we note that in the recent literature, the effect of bubbles on the activation overpotential is occasionally incorrectly identified as the change in active surface area *A* (oftentimes referred to as ECSA in electrocatalysis) in the Butler–Volmer equation describing the current, *i* (eq 2)
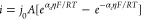
2where *j*_0_ is the exchange current density, α_*i*_ are the transfer coefficients, *F*, *R* and *T* are the Faraday constant, the gas constant and temperature, respectively, and η is the overpotential. However, a true effect on the activation overpotential would mean the change in free energy of adsorbed intermediates, essentially changing the exchange current density *j*_0_ rather than *A*. While changing the active surface area *A* or *j*_0_ has exactly the same quantitative effect on the polarization curves and impedance spectra, the effects on voltammetry are quite different. Since bubbles can really only affect the active surface area *A*, the simulation in Figure 3a-c was performed for different coverages of the electrode with bubbles, where the bubbles progressively reduce the available active surface area. In this case, a decrease in *A* or ECSA, which is proportional to the area of the voltammetric peak at 0.87 V, should be clearly noticeable at higher bubble coverage, as can be seen in Figure 3a. Moreover, for any decrease in ECSA, a proportional decrease in *i* is expected, marked by an apparent shift of the polarization curves to higher overpotentials (Figure 3b). Note, however, that no change in the slope is observed, in contrast with the effect of uncompensated resistance. Finally, the impedance spectra demonstrate a broadening of the semicircle associated with charge-transfer resistance *R*_*ct*_, while the value of the high-frequency intercept of the *x*-axis, attributed to uncompensated resistance does not change. Although all three simulations of the potential impact of reduced active surface area due to bubble formation are in contrast to our experimental results, they do offer a simple tool to identify their potential impact on the “activation overpotential” or more precisely on the ECSA.

Similarly, the simulated responses in the case of concentration overpotential are demonstrated in Figures 3d-f (Supplementary Note 3). We stress that any measurable effect of the concentration overpotential on the polarization curve due to gaseous products at the interface can only be observed for Nernstian systems close to equilibrium potential where the contribution of the backward reaction is significant (blue curve in Figure 3e). The expected effect would be a decrease in reaction rate (current *i*) as well as in the Tafel slope. However, no effect exists at higher overpotentials or for any irreversible systems, which includes OER on Ir and HER on Ni (red curve in Figure 3e), used in our study. Moreover, no measurable effect is expected on the adsorption properties of the electrode indicated by no change in the pseudocapacive currents of the voltammogram (Figure 3d). Finally, as shown in Figure 3f, a significant contribution of the concentration overpotential to the impedance of the system should lead to the appearance of the so-called Warburg impedance, which manifests itself as a 45-degree line at low frequencies in the complex plane plot. The greater the concentration overpotential, the longer this line is in the given frequency range. In the present measurements (Figure 1d), however, no such 45-degree line was detected, which means that the concentration overpotential in this system is insignificant. The simulations shown in Figure 3 are explained in more detail in Supplementary Note 3.

**Figure 4:**
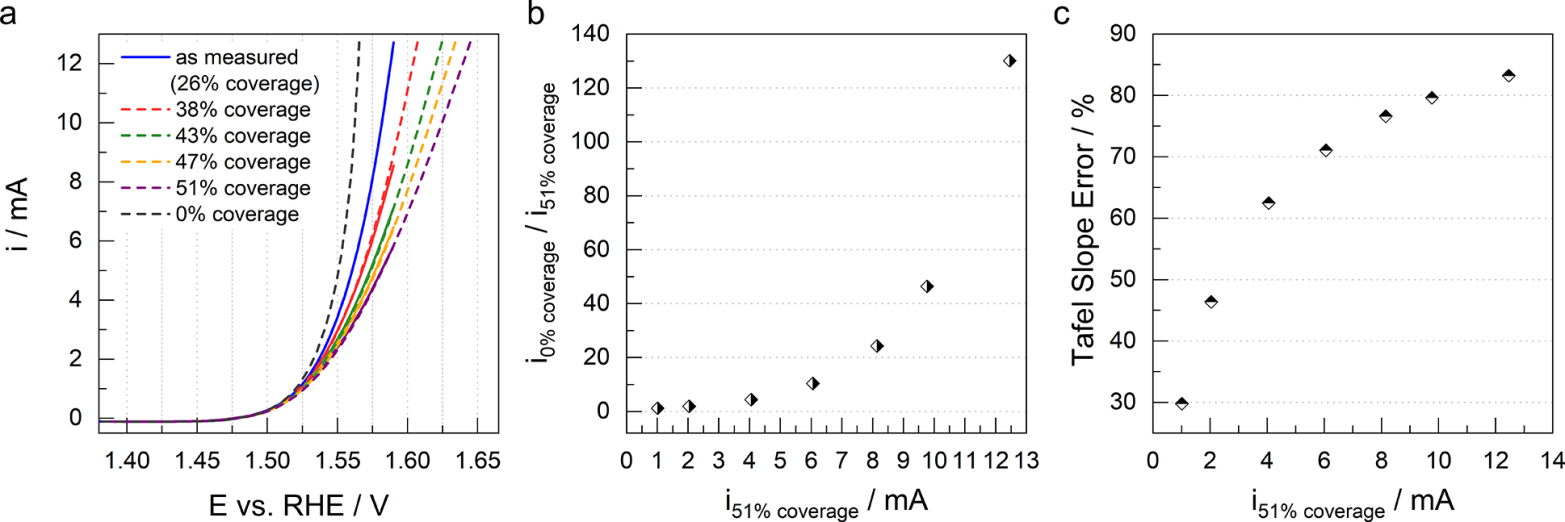
a) Measured (full lines, 95% *iR*-drop compensation) and simulated (dashed lines) polarization curves obtained by applying *R*_*u*_ values obtained from impedance simulation. Black dashed line represents the accurate polarization curve, i.e. by applying full *iR*_*u*_ compensation. b) Ratio between current at 0% and 51% bubble coverage, showing more than 2 orders of magnitude misevaluation of the catalyst’s activity if the effect of bubbles on the *R*_*u*_ is neglected in the analysis, and c) error in determined Tafel slope with increasing uncorrected current (51% surface coverage).

Having established experimentally that the predominant, if not sole, effect of bubbles on the evolution reactions in RDE setup comes from the increase in the uncompensated resistance, we now focus on identifying and quantifying the consequences these bubbles have on the analysis of electrochemical gas evolving reactions. We note that any uncompensated resistance will inevitably lead to errors in activity and Tafel slope determination for any electrochemical reaction. Due to technical limitations of applying an automatic 100% correction of ohmic drop by modern potentiostats using a positive feedback loop,^18^ a lesser compensation (e.g., 85–95%) is usually recommended. Unfortunately, misuse of this recommendation is too often observed in the literature, resulting in random degrees of in situ compensation without any consideration of the additional corrections to 100% after the measurement that must be applied.^19^ As shown above, bubbles create an additional hurdle in applying the 100% resistance compensation because: a) they are formed during the measurement and their effect cannot yet be taken into account, and b) because the impedance is normally measured before the measurement and thus the added resistance is not observed. To show the profound effect of this uncompensated resistance, we again plot the experimentally measured polarization curves (shown in Figure 1a, measured with 95% *iR*-drop compensation and without rotation) for different bubble coverages and also overlay the simulated curves, extending the potential window to 1.65 V (Figure 4a). The errors in the measured activity and Tafel slope with respect to the uncorrected current are given in Figures 4b and c. As can be seen, even a small uncompensated resistance can have a huge impact on the analysis of the electrocatalytic properties. In our OER experiments on the Ir disk, the 51% bubble coverage caused an additional uncompensated resistance of 6 Ω. At 10 mA (51% surface coverage), this caused a deviation of almost 2 orders of magnitude between the measured and the correct activity of the catalyst. At 40 mA, a much smaller uncompensated resistance would have a similar effect. If we erroneously attribute these effects to activation overpotential, we quickly obtain values of 20 mV or more, which are considered significant for activity determination in electrocatalysis, as well as Tafel slopes that are 10 or more mV higher than the actual values. Such results are commonly observed in the electrochemical literature, which lead to false conclusions about the activity, stability or selectivity trends of the electrocatalysts for gas-evolving reactions as well as negatively affect the understanding of the mechanisms of electrocatalytic reactions based on Tafel analysis. Perhaps even a greater concern is that underestimating the effect and magnitude of *R*_*u*_ on the measured current could lead researchers to incorrectly attribute the measured trends to new phenomena e.g. external field effects on the OER catalysis, a recent hot topic in electrocatalysis. These effects on the overpotential and the behavior of the polarization curves, fall well within the realm of small uncompensated resistance, with small overpotential changes observed predominantly at high currents and a change in slope rather than an apparent shift of the polarization curve.

Despite the complexity of challenges that bubbles create for the analysis of gas-evolving catalytic materials, their mitigation for the RDE setup is quite straightforward. The first approach is to try to avoid the accumulation of bubbles altogether. In our experience, this can be achieved with rotation rates above 3600 rpm. The second approach is to correct for the uncompensated resistance for each point after recording the polarization using the bubble coverage obtained during the measurement and the model described above. As shown in Figure 4a, both approaches yield the correct values and slope of the polarization curve, identical to a bubble-free surface. An additional mitigation strategy is also the use of dynamic compensation mode, provided that the chosen potentiostat supports this option. While this approach will significantly reduce the error, it will nevertheless not completely eliminate it.

## Conclusion

In summary, while much has been written about the effects of *R*_*u*_ on electrochemical systems,^15,20^ unfortunately, the electrocatalytic community still does not seem to have completely embraced the extent of its impact on the experimental results. With the help of carefully controlled experiments and simulations, discussed above, we identified and evaluated several possible contributions to the overall measured overpotential that may arise due to the presence of bubbles on the surface of the investigated gas-evolving reaction catalysts. We showed that the observed trends in the activity of our model systems are mainly a result of the increased electrolyte resistance, which is generally assumed to be of constant value. The measured EIS spectra and the use of an innovative two-dimensional transmission line model allowed us to quantitatively estimate the effect of the accumulated bubbles on both the electrolyte and reaction resistance, with the uncompensated electrolyte resistance being the primary reason for the observed trends in the measured polarization curves. We demonstrated that the effect of bubbles on gas-evolving reactions can be effectively controlled through correct *iR*-drop compensation. However, its dynamic nature must be taken into account, which can be done by correcting the measured current point by point with the calculated *R*_*u*_ values for known bubble surface coverages. Furthermore, the effect of bubbles on *R*_*u*_ can be greatly minimized by the employment of the efficient rotation of the electrode, which not only dismisses the hesitations toward the use of RDE for fundamental studies of such reactions but even encourages it. We hope that our contribution will inspire researchers to monitor the changes of *R*_*u*_ when studying gas evolution reactions more thoughtfully and apply this knowledge to the results before making any conclusions on the mechanisms or various effects on electrocatalysis. We believe that only such an approach can lead to correct conclusions and the advancement of our fundamental understanding of these very important reactions.

## Supporting Information Available

Protocols used for the electrode preparation and electrochemical experiments, additional discussion on the results obtained on Ir/C thin film and polycrystalline Ni disk, discussion on the two-dimensional transmission line model and simulations of the polarization curves and impedance spectra for the effect of concentration overpotential, and link to videos showing the formation of bubbles on polycrystalline Ir disk during the electrochemical experiments (PDF)

## References

[ref1] Cherevko S.; Katsounaros I. And yet It Rotates!. Nat. Catal. 2024, 7 (1), 10–11. 10.1038/s41929-023-01100-5.

[ref2] Bashkatov A.; Park S.; Demirkır Ç.; Wood J. A.; Koper M. T. M.; Lohse D.; Krug D. Performance Enhancement of Electrocatalytic Hydrogen Evolution through Coalescence-Induced Bubble Dynamics. J. Am. Chem. Soc. 2024, 146 (14), 10177–10186. 10.1021/jacs.4c02018. 38538570 PMC11009962

[ref3] Park S.; Liu L.; Demirkır Ç.; van der Heijden O.; Lohse D.; Krug D.; Koper M. T. M. Solutal Marangoni Effect Determines Bubble Dynamics during Electrocatalytic Hydrogen Evolution. Nat. Chem. 2023, 15 (11), 1532–1540. 10.1038/s41557-023-01294-y. 37563325

[ref4] van der Heijden O.; Park S.; Eggebeen J. J. J.; Koper M. T. M. Non-Kinetic Effects Convolute Activity and Tafel Analysis for the Alkaline Oxygen Evolution Reaction on NiFeOOH Electrocatalysts. Angew. Chemie - Int. Ed. 2023, 62 (7), 1–9. 10.1002/anie.202216477. PMC10108042 36533712

[ref5] Jovanovič P.; Stojanovski K.; Bele M.; Dražić G.; Koderman Podboršek G.; Suhadolnik L.; Gaberšček M.; Hodnik N. Methodology for Investigating Electrochemical Gas Evolution Reactions: Floating Electrode as a Means for Effective Gas Bubble Removal. Anal. Chem. 2019, 91 (16), 10353–10356. 10.1021/acs.analchem.9b01317. 31379155 PMC6748558

[ref6] Zeradjanin A. R.; Narangoda P.; Spanos I.; Masa J.; Schlögl R. How to Minimize Destabilizing Effect of Gas-Bubbles on Water Splitting Electrocatalysts ?. Curr. Opin. Electrochem. 2021, 30, 100797 10.1016/j.coelec.2021.100797.

[ref7] Chen Q.; Luo L. Correlation between Gas Bubble Formation and Hydrogen Evolution Reaction Kinetics at Nanoelectrodes. Langmuir 2018, 34 (15), 4554–4559. 10.1021/acs.langmuir.8b00435. 29569923

[ref8] Fathi Tovini M.; Hartig-Weiß A.; Gasteiger H. A.; El-Sayed H. A. The Discrepancy in Oxygen Evolution Reaction Catalyst Lifetime Explained: RDE vs MEA - Dynamicity within the Catalyst Layer Matters. J. Electrochem. Soc. 2021, 168 (1), 014512 10.1149/1945-7111/abdcc9.

[ref9] Lazaridis T.; Stühmeier B. M.; Gasteiger H. A.; El-Sayed H. A. Capabilities and Limitations of Rotating Disk Electrodes versus Membrane Electrode Assemblies in the Investigation of Electrocatalysts. Nat. Catal. 2022, 5 (5), 363–373. 10.1038/s41929-022-00776-5.

[ref10] El-Sayed H. A.; Weiß A.; Olbrich L. F.; Putro G. P.; Gasteiger H. A. OER Catalyst Stability Investigation Using RDE Technique: A Stability Measure or an Artifact?. J. Electrochem. Soc. 2019, 166 (8), F458–F464. 10.1149/2.0301908jes.

[ref11] Hansen J. N.; Prats H.; Toudahl K. K.; Mørch Secher N.; Chan K.; Kibsgaard J.; Chorkendorff I. SI Is There Anything Better than Pt for HER?. ACS Energy Letters. 2021, 6, 1175–1180. 10.1021/acsenergylett.1c00246. 34056107 PMC8155388

[ref12] Garcés-Pineda F. A.; Blasco-Ahicart M.; Nieto-Castro D.; López N.; Galán-Mascarós J. R. Direct Magnetic Enhancement of Electrocatalytic Water Oxidation in Alkaline Media. Nat. Energy 2019, 4 (6), 519–525. 10.1038/s41560-019-0404-4.

[ref13] Angulo A.; van der Linde P.; Gardeniers H.; Modestino M.; Fernández Rivas D. Influence of Bubbles on the Energy Conversion Efficiency of Electrochemical Reactors. Joule 2020, 4 (3), 555–579. 10.1016/j.joule.2020.01.005.

[ref14] Zhao X.; Ren H.; Luo L. Gas Bubbles in Electrochemical Gas Evolution Reactions. Langmuir 2019, 35 (16), 5392–5408. 10.1021/acs.langmuir.9b00119. 30888828

[ref15] Zheng W. IR Compensation for Electrocatalysis Studies: Considerations and Recommendations. ACS Energy Lett. 2023, 8 (4), 1952–1958. 10.1021/acsenergylett.3c00366.

[ref16] Ardizzone S.; Fregonara G.; Trasatti S. Inner” and “Outer” Active Surface of RuO2 Electrodes. Electrochim. Acta 1990, 35 (1), 263–267. 10.1016/0013-4686(90)85068-X.

[ref17] Frazer E. J.; Woods R. The Oxygen Evolution Reaction on Cycled Iridium Electrodes. J. Electroanal. Chem. 1979, 102 (1), 127–130. 10.1016/S0022-0728(79)80036-4.

[ref18] Oelßner W.; Berthold F.; Guth U. The IR Drop - Well-Known but Often Underestimated in Electrochemical Polarization Measurements and Corrosion Testing. Mater. Corros. 2006, 57 (6), 455–466. 10.1002/maco.200603982.

[ref19] Heenan A. R.; Hamonnet J.; Marshall A. T. Why Careful IR Compensation and Reporting of Electrode Potentials Are Critical for the CO2Reduction Reaction. ACS Energy Lett. 2022, 7 (7), 2357–2361. 10.1021/acsenergylett.2c00800.

[ref20] Son Y. J.; Marquez R. A.; Kawashima K.; Smith L. A.; Chukwuneke C. E.; Babauta J.; Mullins C. B.; Navigating Navigating iR Compensation: Practical Considerations for Accurate Study of Oxygen Evolution Catalytic Electrodes. ACS Energy Lett. 2023, 8 (10), 4323–4329. 10.1021/acsenergylett.3c01658.

