# Supplementary material for: Bubble Trouble: Quantifying the Effects of Bubbles on the Electrochemical Interface
Source: ACS Catal. 2025 Apr 4;15(8):6380–5. doi: 10.1021/acscatal.5c00144 (PMC12012731; doi:10.1021/acscatal.5c00144)
Supplement: Supplementary file 1 — cs5c00144_si_001.pdf [file cs5c00144_si_001.pdf]

Supporting Information

Bubble Trouble: Quantifying the Effects of Bubbles on the Electrochemical Interface

Anja Logar [a,b] Dževad K. Kozlica,[a,c] Ožbej Vodeb,[a,d] Miran Gaberšček,[a,e] Nejc Hodnik [a,b,f] and Dušan Strmčnik [a]*

[a] National Institute of Chemistry, Department of Materials Chemistry, Hajdrihova 19, 1000 Ljubljana, Slovenia

[b] University of Nova Gorica, Graduate School, Vipavska 13, 5000 Nova Gorica, Slovenia

[c] University of Maribor, Faculty of Chemistry and Chemical Engineering, Smetanova ulica 17, 2000 Maribor, Slovenia

[d] Jozef Stefan International Postgraduate School, Jamova 39, 1000 Ljubljana, Slovenia

[e] University of Ljubljana, Faculty of Chemistry and Chemical Technology, Večna pot 113, 1000 Ljubljana, Slovenia

[f] Institute of Metals and Technology, Department of Physics and Chemistry of Materials, Lepi Pot 11, 1000 Ljubljana, Slovenia

** corresponding author: dusan.strmcnik@ki.si*

KEYWORDS: oxygen evolution reaction, hydrogen evolution reaction, bubbles, rotating disk electrode, uncompensated resistance.

Methods

Electrode preparation

Ir polycrystalline disk (Mateck) was polished with a 1 µm diamond paste and thoroughly cleaned by rinsing and sonicating in Milli-Q water for 15 minutes with changing the water every three minutes. The cleaned disk was protected with a drop of water and embedded in the rotating disk electrode holder. The electrode with the powdered sample was prepared by dropping 20 µL of ink onto the glassy carbon disk, which was previously polished with 1 µm alumina slurry and cleaned by using the same sonication protocol as described above. The ink was prepared by dispersing 20 % Ir on Vulcan XC-72 (PREMETEK), Ir/C in Milli-Q water (18.2 MΩ cm). Nafion solution (D520, Ion Power) was added at a ratio of 1:4 between ionomer and catalyst, and the pH of the ink was adjusted to 11 by adding 1M KOH solution (Titripur, Supelco). The final loading of the catalyst on the disk was 100 µg cm^{-2}. The Ni polycrystalline electrode was prepared by inductive heating for 10 min at ~1270 K in an argon hydrogen flow (3% hydrogen). The annealed specimens were cooled slowly to room temperature under an inert atmosphere and immediately covered with a droplet of Milli-Q water. The electrode was then assembled into a rotating disk electrode (RDE) setup. All gasses used in the experiments were 5N5 quality and purchased from Messer.

Electrochemical experiments

The measurements were carried out in a standard three-electrode glass cell with an EmStat4X (PalmSens) potentiostat. All potentials are reported vs. reversible hydrogen electrode (RHE). The electrolytes used were 0.1M $HClO_4$ (70% Rotipuran Supra, Carl Roth) and 0.1M KOH (99.995% Suprapur, Supelco) and were always purged with Ar prior to the experiment. Polycrystalline Ir and powdered Ir-covered rotating disk electrodes were used as working electrodes for OER measurements in acidic media and polycrystalline Ni disk was used as a working electrode for HER in alkaline media. The reversible HydroFlex hydrogen electrode (Gaskatel GmbH) was used as the reference electrode. For experiments involving Ir, a glassy carbon rod was employed as the counter electrode, whereas for experiments involving Ni, a nickel wire was used. The Ir-based working electrodes were immersed in the electrolyte at 0.05 V, and the experiment was started by growing hydrous oxide on the Ir surface by electrochemical cycling between 0.05 and 1.45 V (300 cycles, 1 V/s). After surface oxide formation, a slow voltammogram (50 mV/s) from 0.4 to 1.59 V with an 95% R_u compensation was recorded. The EIS spectra was measured in the following step at 1.52 V in the range from 100 kHz and 1 Hz with an amplitude of 10 mV and 10 frequency points per decade. To measure the R_u without the presence of bubbles on the surfaces of all three electrodes, the EIS was measured at potential below the onset of OER, i.e. 1.3 V. For the experiments on Ni, the electrode was immersed at 0.0 V in an Ar-saturated 0.1 M KOH solution. Cyclic voltammograms were recorded to simultaneously measure the electrochemically active surface area and the hydrogen evolution reaction (HER) activity. The voltammetric scans began with a positive sweep from 0.0 V to 0.565 V, followed by a negative sweep

down to −0.435 V, at a scan rate of 50 mV s^{-1}. After each voltammetry measurement, the corresponding EIS spectra were recorded at −0.315 V, covering a frequency range from 100 kHz to 10 Hz with an amplitude of 10 mV and 10 frequency points per decade.

Images and videos of evolved bubbles on the surface of the disk as a result of electrochemical reaction were recorded using a Media-tek MT4096 USB microscope.

Surface coverages by bubbles were estimated using an ImageJ program.

Results

The supplementary videos for the electrochemical experiments on polycrystalline Ir disk (Figure 1) can be downloaded from the following GitHub repository: https://github.com/ECatGroup/bubble_trouble

Supplementary Note 1:

1.1 OER on a thin film of Ir/C in 0.1M $HClO_4$

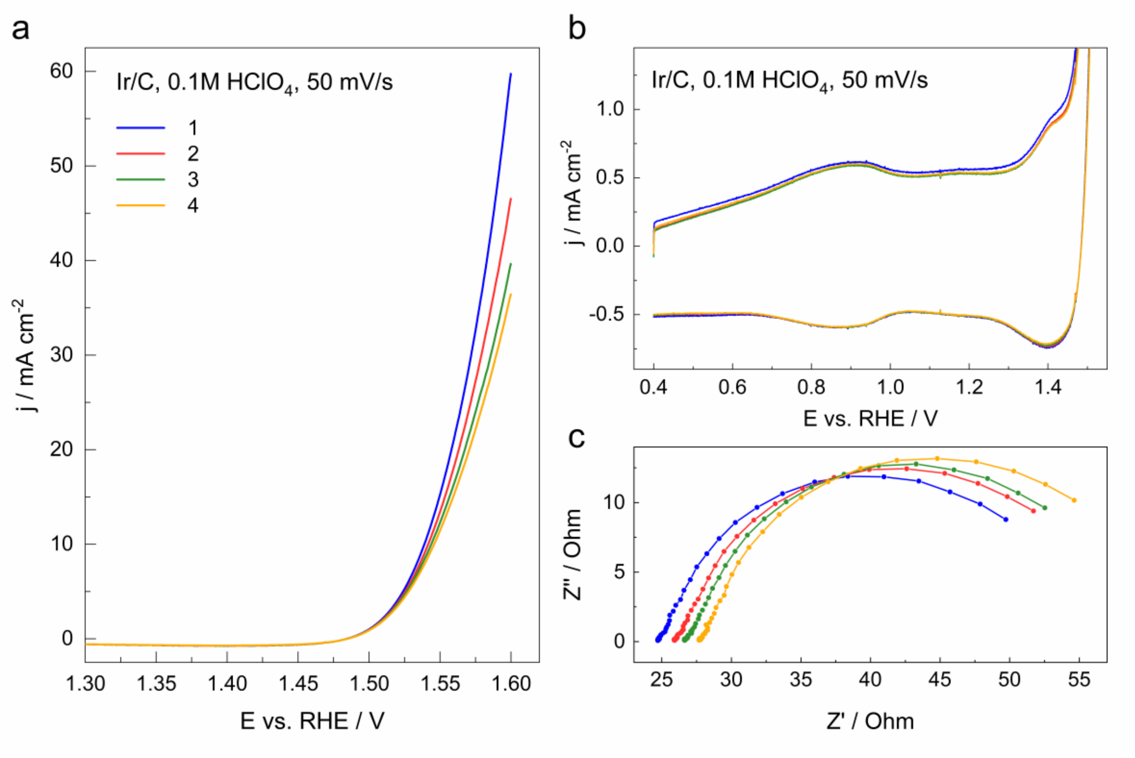

Figure S1: Electrochemical results obtained on a thin film of Ir/C deposited on glassy carbon RDE in 0.1M $HClO_4$. a) Consecutive OER polarization curves (cathodic scans) with increasing bubble surface coverage, measured with scan rate 50 mV/s, b) cyclic voltammograms indicating no change in the available surface area of the electrode with increasing bubble surface coverages, measured with scan rate 50 mV/s, and c) EIS spectra recorded at 1.52 V after each cyclic voltammogram.

Images of the glassy carbon disk with a thin film of Ir/C catalyst and iR_u-drop correction:

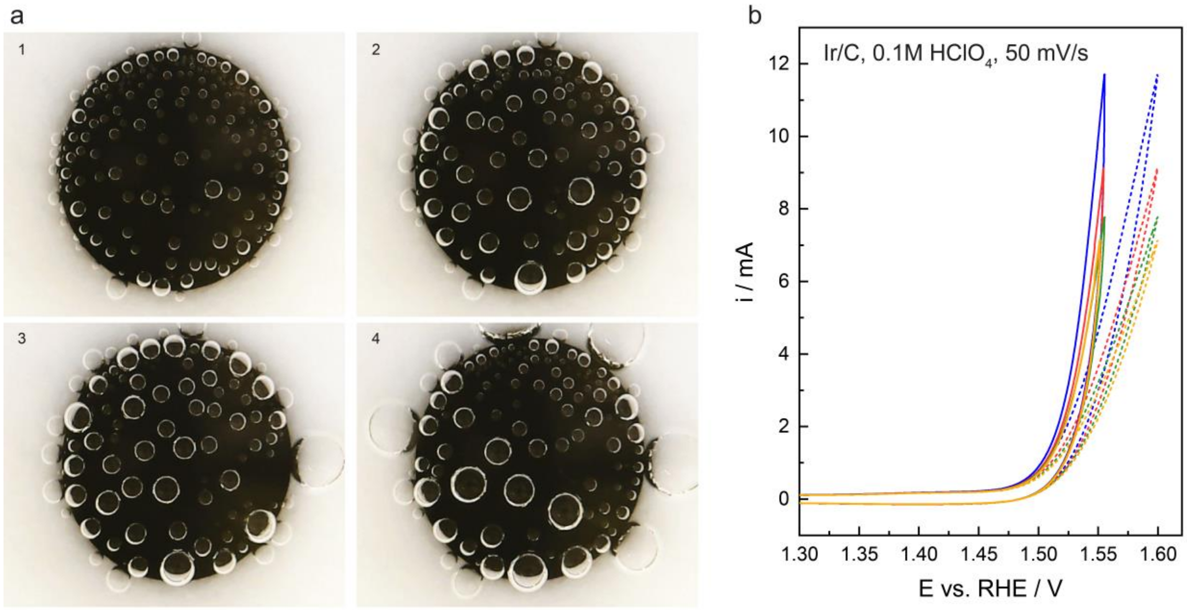

Figure S2: a) Micrographs of the glassy carbon disk with a thin film of Ir/C catalyst with different surface coverages taken after each CV. The estimated surface bubble coverage values are 23% after cycle 1, 32% after cycle 2, 35% after cycle 3, and 37% after cycle 4; b) iR_u-drop corrected OER polarization curves demonstrating the magnitude of the effect of bubbles on the increase of the uncompensated resistance.

1.2 HER on polycrystalline Ni disk in 0.1M KOH

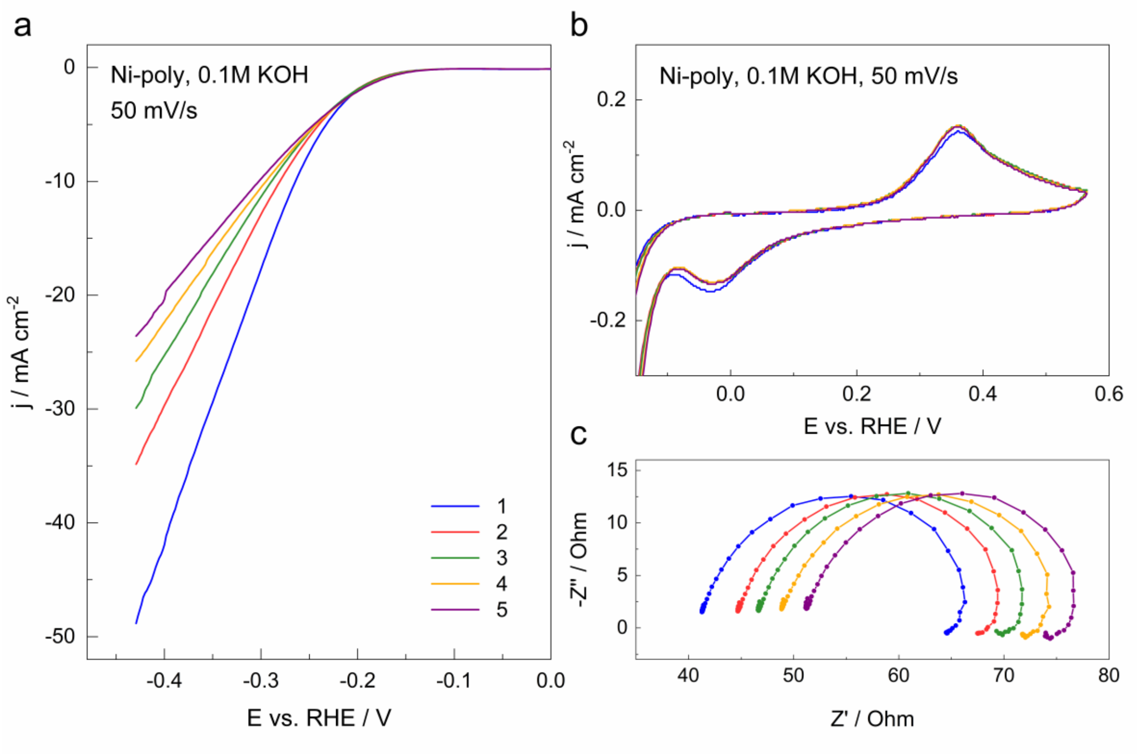

Figure S3: Electrochemical results obtained on polycrystalline Ni RDE, measured in 0.1M KOH; a) Consecutive HER polarization curves with increasing bubble surface coverage, recorded with a scan rate 50 mV/s b) cyclic voltammograms indicating the changes in the available surface area of the disk measured with a scan rate 50 mV/s and c) EIS spectra measured at -0.315V on the disk with varying bubble surface coverage.

To test the universality of our approach, we performed the same investigation as on polycrystalline Ir, in detail discussed in the main part of the article, on two additional platforms. First, we again measured OER in acidic media but used a different working electrode, namely a thin film of a powdered Ir-based catalyst, Ir/C deposited on a glassy carbon RDE. A similar electrode has been used in a recent publication, questioning the suitability of RDE for the measurement of the stability of OER catalysts, due to the accumulation of bubbles in the catalyst layer.[1] Our results show that bubbles similarly to polycrystalline Ir significantly affect only the resistance of the electrolyte. By correctly compensating for the additional R_u increase due to the accumulation of macrobubbles, the polarization curves overlap entirely (Figure S2b). Additional confirmation is the overlap of the voltammetries (Figure S1b), used for the estimation of electrochemically active surface area, which shows that, even if present, microbubbles do not have a significant effect on the decrease of active surface area and the measured polarization curves.

In the second example, our conclusions were tested on a polycrystalline Ni surface, but with a different reaction, i.e. HER and electrolyte, i.e. 0.1M KOH solution. Based on the results, presented in Figure

S3, which again give the same trends of the measured polarization curves, voltammetries[2] and EIS spectra as described in the main part of the article, we demonstrate that our approach can be universally applied to any gas-evolving reaction, measured with an RDE setup.

Supplementary Note 2:

Two-dimensional model for simulating the influence of bubbles on the impedance spectrum of a flat electrode in contact with a liquid electrolyte

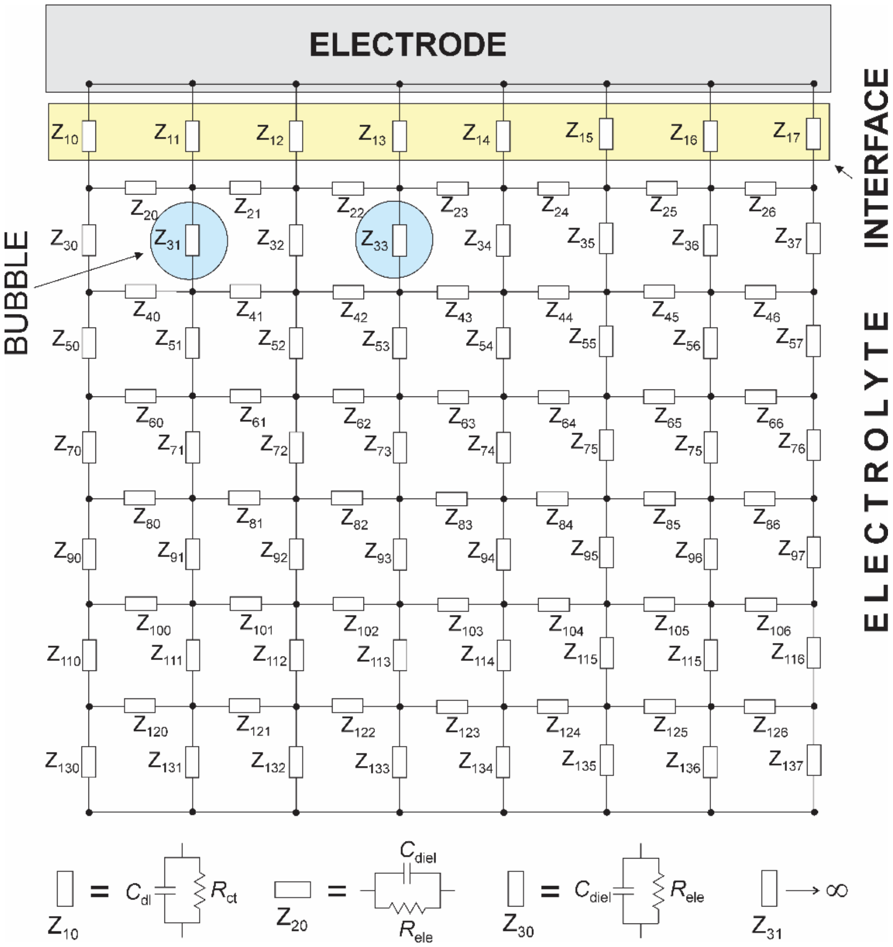

Figure S4: Two-dimensional transmission line model describing the effect of bubbles on the impedance behavior of an electrode in contact with the electrolyte containing bubbles. The pure electrolyte is described by elements containing a parallel combination of the electrolyte resistance (R_{ele}) and its capacitance, which arises due to the dielectric properties of the electrolyte (C_{diel}). The interface between electrode and electrolyte is described by elements containing a parallel combination of the charge transfer resistance (R_{ct}) and the double layer capacitance (C_{dl}). The impedance of the bubbles is assumed to be infinite. Examples of simulations using the proposed transmission line model are shown and commented in the main article.

The underlying principle of the present simulations is the construction of a physics-based transmission line to describe the impedance response of an electrochemical system.[3] Normally, such transmission

line models are one-dimensional, i.e. they describe transport/reaction phenomena in the direction perpendicular to the electrode surface (usually referred to as direction *x*). However, such one-dimensional models are not able to account for inhomogeneities in the *y*- and/or *z*-direction, which are parallel to the electrode surface. An example of such inhomogeneities are bubbles, which create regions where no electrical conduction/reaction can take place. If we assume that the distribution of inhomogeneities (bubbles) is statistically similar in the *y*- and *z*-directions, a two-dimensional model should be sufficient to describe their effect(s).

The present transmission line model is constructed by dividing the electrolyte in contact with the electrode into regions that are equal to or smaller than the typical size of the bubbles (Figure S4). The impedance of each region is then described by a corresponding electrical element (Z_i). A similar procedure has recently been used to describe the effects of the quality of contact at the interface between the electrode and solid electrolyte on the impedance response.[4] Specifically, due to the finite conductivity of the electrolyte, the regions containing pure electrolyte are described with resistors and capacitors representing the geometric capacitance of the electrolyte (due to its dielectric properties). The regions containing pure bubbles are described with an infinite impedance, so it is assumed that there is no electrical transport through the bubbles. The regions at the interface between the electrode and the electrolyte are described by charge transfer resistances parallel to double layer capacitors.

Before applying the model to explain the present results, we performed a comprehensive sensitivity analysis by systematically varying the values of model parameters such as electrolyte resistance, charge transfer resistance and, in particular, the geometry and position of the bubbles. Finally, we collected the actual geometry data (average size and average geometric distribution of the bubbles, size of the electrode and distance between the working and reference electrodes). Based on the collected data, we simulated the curves shown in the main article (Figure 2b and c). It is important to note that the shape of the theoretical curve in Figure 2c is completely determined by the bubble geometry (which we adopted from the experimental observation), while the vertical position depends on the electrolyte resistance. Since we did not know the exact value of the electrolyte resistance (due to uncertainties in the cell geometry), we decided to position the theoretical curve so that the first theoretical value (at zero bubble coverage) coincides with the measured value of the electrolyte resistance at this condition. All other points are then calculated from the model and the average geometry of the bubbles. In other words, the method used did not involve curve fitting.

Supplementary Note 3:

3.1 Simulation of a concentration overpotential effects on HER polarization curves

The simulation package used was ElectroKitty, a free Python script found on Git Hub,[5] which allows simulation of the current response of various mechanisms, given the potential program. For the electrochemical response, the parameters α, k_0 and $E^{0'}$, were 0.5, $0.5\cdot10^3$ ms^{-1}, -0.1 V respectively, for the Volmer step the constants were both set to 10^{12}. The initial active site density, electroactive surface area, proton concentration and diffusion constant were all set to: $0.75\cdot10^{-7}$ $molm^{-2}$, 10^{-4} m^2, 100 $molm^{-3}$ and 10^{-8} m^2s^{-1} respectively.

To simulate the effect of the concentration overpotential on the polarization curves shown in the main article (Figure 3e), we used the Volmer-Tafel mechanism given by equations S1 and S2. The only difference in simulations of reversible and irreversible reaction is that the back reaction in the first case is allowed, while in the second it is not.

$$H^+_{aq} + * \rightleftharpoons H^* \qquad (S1)$$

$$2H^* \rightleftharpoons 2* + H_{2,aq} \qquad (S2)$$

3.2 Simulation of the effect of activation and concentration overpotential on the EIS spectra

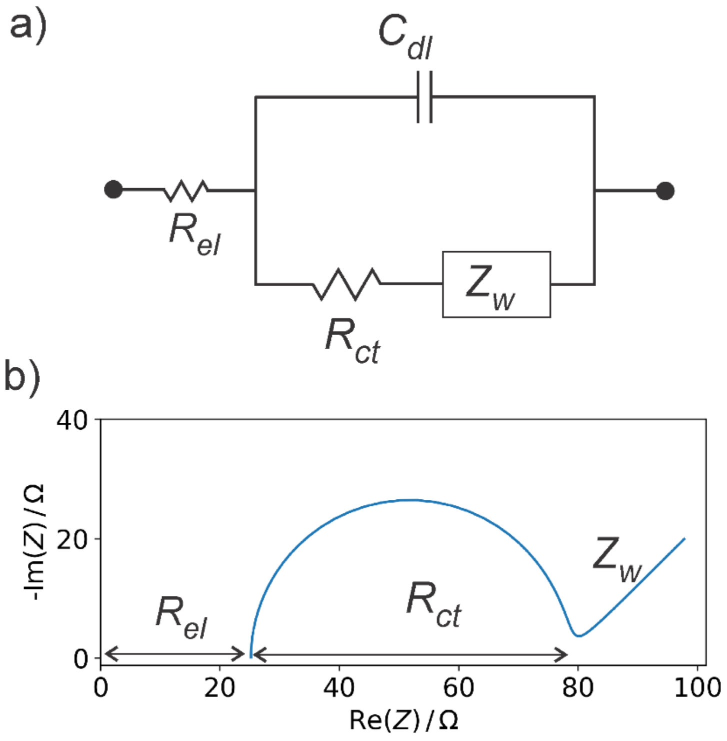

Figure S5: a) Randles equivalent circuit and b) its typical impedance response plotted in the complex plane. R_{el}, R_{ct} and Z_w denote the electrolyte resistance, the charge transfer resistance and the Warburg impedance, respectively.

To explain the general effects of activation and concentration overpotential on the EIS spectra, we use the textbook model known as the Randles equivalent circuit (Fig. S5).[6] The Randles equivalent circuit is derived assuming the migration and diffusion of redox-active species in the electrolyte to and away from the electrode where the corresponding redox reaction takes place. The latter is described by the Butler-Volmer equation, which is seen as the charge transfer resistance (R_{ct}) in the impedance spectrum. The migration of charged redox species in the electrolyte is detected as the electrolyte resistance (R_{el}), while the diffusion of these species at low frequencies is visible as Warburg impedance, Z_w. An increase in electrolyte resistance is reflected in a proportional increase in the value of R_{el}. An increase in overvoltage overpotential is reflected in a proportional increase in the value of R_{ct}. Similarly, an increase in concentration overpotential is seen as an increase in the magnitude (length) of the line denoted as Z_W. Importantly, Z_w is not detected at all in the present system, strongly suggesting that the contribution of concentration overpotential to the impedance spectra is negligible – for all measurements shown in Figures 1d, S1c and S3c.

References

(1) El-Sayed, H. A.; Weiß, A.; Olbrich, L. F.; Putro, G. P.; Gasteiger, H. A. OER Catalyst Stability Investigation Using RDE Technique: A Stability Measure or an Artifact? *J. Electrochem. Soc.* **2019**, *166* (8), F458–F464. https://doi.org/10.1149/2.0301908jes.

(2) Beden, B.; Floner, D.; Léger, J. M.; Lamy, C. A Voltammetric Study of the Formation on Hydroxides and Oxyhydroxides on Nickel Single Crystal Electrodes in Contact with an Alkaline Solution. *Surf. Sci.* **1985**, *162* (1–3), 822–829. https://doi.org/10.1016/0039-6028(85)90985-9.

(3) Jamnik, J.; Maier, J. Generalised Equivalent Circuits for Mass and Charge Transport: Chemical Capacitance and Its Implications. *Phys. Chem. Chem. Phys.* **2001**, *3* (9), 1668–1678. https://doi.org/10.1039/b100180i.

(4) Eckhardt, J. K.; Klar, P. J.; Janek, J.; Heiliger, C. Interplay of Dynamic Constriction and Interface Morphology between Reversible Metal Anode and Solid Electrolyte in Solid State Batteries. *ACS Appl. Mater. Interfaces* **2022**, *14* (31), 35545–35554. https://doi.org/10.1021/acsami.2c07077.

(5) ElectroKitty Git Hub page https://github.com/RedrumKid/ElectroKitty.

(6) Barsoukov, E.; Macdonald, J. R. *Impedance Spectroscopy: Theory, Experiment, and Applications*, 2nd Editio.; Barsoukov, E., Macdonald, J. R., Eds.; John Wiley & Sons, Inc.: Hoboken, NJ, USA, 2005. https://doi.org/10.1002/9781119381860.
